# COVID-19 in Morocco: Nurses’ Knowledge of Anti-COVID-19 Vaccines and Their Involvement in Vaccine Vigilance

**DOI:** 10.3390/tropicalmed10040097

**Published:** 2025-04-06

**Authors:** Fatima Zahra Laamiri, Manar Aarrad, Abdelmounaim Manoussi, Youssef Baba Khouya, Fatine Hadrya, Mohamed Chahboune, Amina Barkat

**Affiliations:** 1 Health Sciences and Technology Laboratory, Higher Institute of Health Sciences of Settat, Hassan First University of Settat, Settat 26000, Morocco; m.aarrad@uhp.ac.ma (M.A.); abdelmounaim.manoussi@um6p.ma (A.M.); youssef.babakhouya@uhp.ac.ma (Y.B.K.); fatine.hadrya@uhp.ac.ma (F.H.); mohamed.chahboune@uhp.ac.ma (M.C.); 2 Laboratory of Pharmacology, Neurobiology, Anthropology, Environment and Behavior, Faculty of Science of Semlalia, Cadi Ayad University, Marrakesh 40000, Morocco; 3 Faculty of Medicine, Health and Nutrition Research Team of the Mother-Child Couple, Mohammed V University, Rabat 10100, Morocco; barakatamina@hotmail.fr

**Keywords:** COVID-19 vaccination, knowledge, nursing professionals, post-vaccination adverse effects

## Abstract

The COVID-19 pandemic has highlighted the crucial role of nurses in managing health crises, particularly in implementing vaccination campaigns launched in many countries worldwide. This descriptive study assesses nurses’ knowledge of COVID-19 vaccines and their involvement in vaccine vigilance. Conducted over four months in 2022 among 200 primary healthcare nurses in the Fès-Meknès region, the data were collected using a questionnaire developed and validated by a multidisciplinary team. The results show that 60% of participants self-reported being unaware of the nature of COVID-19 vaccines, and 49.5% did not understand the concept of vaccine pharmacovigilance. Additionally, 76.5% had not received any pharmacovigilance training in this pandemic context, 80% had never been in contact with pharmacovigilance centers, and 48.5% expressed a need for training in this field. One-third of the participants were unaware of severe adverse effects. The most frequently reported adverse effects were fever (76.5%), malaise (73%), and anxiety (63%). These results highlight the importance of strengthening continuous training and improving the coordination among various healthcare sector stakeholders to reduce vaccine hesitancy, enhance healthcare expertise, and ensure the effectiveness of vaccination campaigns during current and future pandemics.

## 1. Introduction

The advent of the pandemic crisis in Morocco has led to the need to adopt numerous measures to control the spread of the virus and reduce infections, hospitalizations, and deaths, including vaccination, which is a key strategy and a powerful tool for defeating this global pandemic and protecting people’s health [1]. Several studies in the scientific literature have demonstrated the protective role of COVID-19 vaccination in reducing the risk of infection and severe outcomes [2,3,4].

Since the start of the pandemic, more than 1,279,450 officially confirmed cases of COVID-19 have been reported in Morocco, resulting in 16,310 officially attributed deaths linked to the virus [5]. In response, the country launched a national vaccination campaign on 28 January 2021 [6]. By 22 November 2024, 24.9 million people had received a first dose, 23.4 million a second, 6.8 million a third, and 61,586 a fourth [5].

However, despite the undeniable benefits of vaccination, vaccine hesitancy remains a significant problem, identified by the World Health Organization (WHO) as one of the world’s top ten health threats [7]. This phenomenon refers to the delay or refusal of vaccines despite their accessibility. Several factors contribute to this reluctance, including concerns about the safety and efficacy of vaccines, distrust of health authorities and the pharmaceutical industry, and the spread of false information on social networks. In the context of the pandemic, the rapid introduction of SARS-CoV-2 vaccines has heightened these concerns by raising questions about their efficacy, safety, and post-vaccination surveillance mechanisms, reinforcing vaccine hesitancy. As a result, nurses continue to play a vital role in improving the uptake of the vaccine, given their involvement in all stages of the campaign, including organization, distribution, and administration [8].

Indeed, the role of the nursing professionals begins before the vaccine is administered with the pre-vaccination assessment, which involves taking a medical history and checking for any contraindications to vaccination, then during administration by providing information to the patient on potential adverse effects and symptoms to watch out for, and finally after administration by advising patients who develop temporary local or systemic reactions and also by reporting adverse events to pharmacovigilance systems for the ongoing monitoring of vaccine safety.

However, studies have shown that gaps in healthcare professionals’ knowledge of vaccines and pharmacovigilance compromise the effectiveness of vaccination programs [9,10]. In this context, this study aims to explore Moroccan nurses’ knowledge of COVID-19 vaccines and their involvement in vaccine vigilance to strengthen continuing education strategies and encourage the population to take up vaccination.

## 2. Materials and Methods

### 2.1. Type and Location of Study

This study aimed to assess nurses’ knowledge of COVID-19 vaccines and pharmacovigilance in the context of the national vaccination campaign. It was designed as a descriptive observational study conducted over four months, from 15 February to 18 June 2022, in primary healthcare facilities in the Fès-Meknès region of Morocco. These facilities were selected due to their active involvement in the national COVID-19 vaccination campaign.

### 2.2. Study Population and Sampling

We used non-probability convenience sampling combined with a snowball approach. We adopted this method because it is difficult to reach the target population directly and requires relying on relational networks to do so.

The investigator initially reached out to healthcare professionals within their network. These contacts, who were actively involved in the field, had easy access to the target population. Paper-based questionnaires were distributed to these contacts, and they were requested to be shared with other members of the target population. This approach helped expand the study’s reach by leveraging the professional and personal networks of the initial participants. The inclusion criteria for the study were being a nursing professional involved in the COVID-19 vaccination campaign in the Fès-Meknès region, Morocco, covering both urban and rural areas, and voluntarily agreeing to participate. The completed questionnaires were then returned to the investigator. Thus, the sample size depended on the number of nursing professionals accessible through these networks who were willing to participate in the study.

The pragmatic and relational approach adopted allowed for (1) the rapid recruitment of a sufficient number of participants despite access constraints and (2) the inclusion of professionals working in rural areas, which are often difficult to reach using conventional sampling methods.

However, selection bias may have been introduced, as the selection was non-random, potentially limiting the generalizability of the results to the target population. This approach was well suited to the field constraints. Despite the limitations of the chosen method, our study provided valuable insights into the experience of nurses involved in the COVID-19 vaccination campaign in the study region.

### 2.3. Data Collection

To achieve the objective of this study, a questionnaire was drawn up based on a literature review and then submitted to a validation process by a panel of experts in the fields of vaccination, pharmacovigilance, and nursing. This validation ensured the questions were relevant, clear, and aligned with the study objectives. The expertise-based approach was chosen because it is commonly used in studies assessing the knowledge of healthcare professionals, particularly when the questionnaire is based on well-established concepts. This tool enabled us to explore the following four aspects:

- General information about the population: age, sex, specialty, seniority, place of practice, and continuing education.
- Information relating to the COVID-19 pandemic and the anti-vaccination platform: participation in training during the COVID-19 pandemic, implementation of systematic alerts for new patients with COVID-19, availability of materials for barrier measures against COVID-19, target population for vaccination, use of different vaccines in health facilities, the most frequently requested vaccines and participation in training on COVID-19 vaccination, the main types of COVID-19 vaccine, knowledge of the nature of the vaccines (AstraZeneca, Moderna, Pfizer), and perception of the health risk of the COVID-19 vaccine.
- Knowledge of pharmacovigilance: knowledge of pharmacovigilance, participation in training on pharmacovigilance in the context of the COVID-19 pandemic, contact with pharmacovigilance centers during the pandemic, and the need for pharmacovigilance training.
- Knowledge of post-vaccine adverse events: commonly reported post-vaccine adverse events, the protocol for management and follow-up of post-vaccine adverse events, perceived deaths attributed to COVID-19 vaccine, perceived safety of mRNA vaccines, perceived risk of cardiac inflammation associated with mRNA vaccines, and perceived involvement of AstraZeneca and Johnson in venous thrombosis and Guillain–Barré syndrome.

To test the questionnaire’s clarity and ease of understanding, a pre-test was carried out with a representative sample of the target population, which comprised 10 people who were excluded from the final study sample. Following the comments received during the pre-test, modifications were made before the final distribution of the questionnaire.

## 3. Results

### 3.1. Demographic and Professional Characteristics of Participants

The demographic and professional data of the participants in this survey are shown in Table 1. The data analysis revealed a preponderance of people aged over 25 and of women, with prevalence rates of 67% and 74%, respectively. The male/female sex ratio was 0.34. The distribution of professionals according to seniority was statistically similar. In contrast, the distribution according to place of practice was dominated by urban areas, which accounted for three-quarters of the participants.

### 3.2. Vaccination Campaign Against COVID-19 and Anti-Vaccination Platform

This study assessed nurses’ knowledge of COVID-19 vaccination. Table 2 shows that 70.5% of the surveyed nurses had received no vaccination training, and 68.5% of their facilities lacked a formal alert system for rapidly reporting new COVID-19 cases. Furthermore, a significant proportion of participants (44%) indicated that their primary healthcare facilities were not equipped with adequate barrier measures against the virus.

Regarding the vaccines available in the studied primary healthcare centers, AstraZeneca (87.5%), Sputnik (80.5%), and Sinopharm (72%) were the most frequently used. However, nearly half of the participants (60%) were unaware of the nature of the administered vaccines, and 57% expressed concerns about potential adverse effects.

### 3.3. Pharmacovigilance and Vaccination Against COVID-19

**Figure 1:**
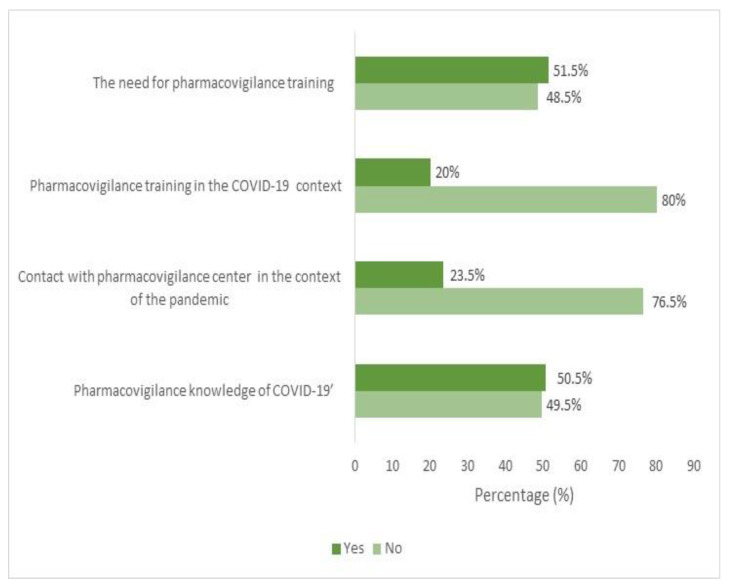
Pharmacovigilance knowledge and training needs among nursing professionals during COVID-19 pandemic.

The analysis of healthcare professionals’ knowledge of pharmacovigilance in COVID-19 (Figure 1) revealed that nearly half of the participants (49.5%) lacked knowledge about COVID-19 pharmacovigilance. Moreover, most (80%) reported no contact with pharmacovigilance centers. Additionally, three-quarters of the participants (76.5%) had not received any training in pharmacovigilance related to the pandemic. However, over half (51.5%) expressed a need for training in COVID-19 pharmacovigilance.

### 3.4. Post-Vaccine Adverse Reactions in the Context of COVID-19

**Figure 2:**
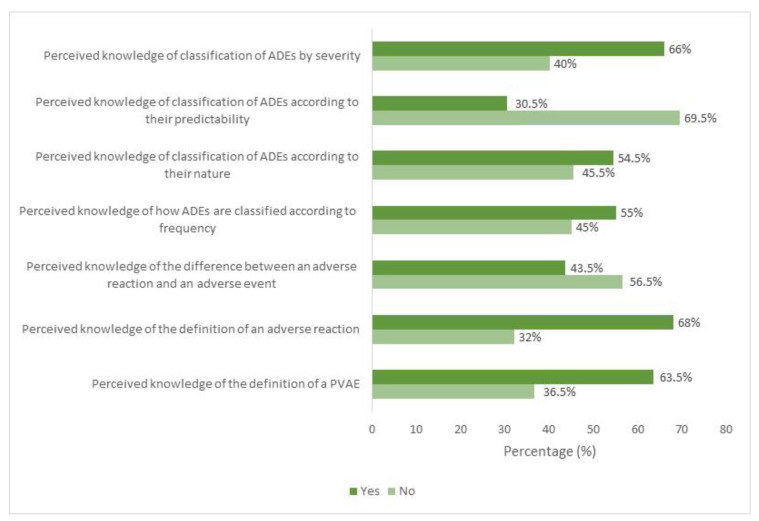
Distribution of Nursing Professionals According to perceived knowledge of Post-Vaccination Adverse Events and Related Parameters. Note: ADE: Adverse Drug Events, PVAEs: Post-Vaccination Adverse Events.

The analysis of the results in Figure 2 highlights significant gaps in the understanding of fundamental concepts related to adverse drug events (ADEs) and post-vaccination adverse events (PVAEs). Although the majority of the nurses surveyed claim to know the definition of PVAEs (63.5%) and adverse reactions (68%), more than half (56.5%) are unable to distinguish between an adverse reaction and an adverse event. Regarding the classification of ADEs, knowledge varies depending on the criteria assessed. While a relative majority grasps the classification based on the frequency (55%) and nature of ADEs (54.5%), the understanding of their classification according to predictability is particularly low (30.5%).

Furthermore, the classification of ADEs by severity is better understood (66%), suggesting a better comprehension of the severity of adverse effects among the nurses surveyed. The most frequently cited severe events by nurses, based on their perceptions and experiences, included intracerebral hemorrhages (69%) and thromboembolic events (58%). Additionally, 34% of nurses reported having encountered cases of myocarditis and pericarditis. However, these reports are based on subjective accounts rather than confirmed clinical diagnoses.

## 4. Discussion

The COVID-19 pandemic highlighted the key role played by nurses in managing health crises, mainly through the vaccination campaign. As key players in providing information and administering vaccines, their level of knowledge has a direct influence on the effectiveness of the prevention strategies implemented. This study focused on a population of nursing professionals practicing in primary healthcare establishments in Morocco to assess their level of knowledge about COVID-19 vaccination and vigilance.

### 4.1. Knowledge and Training of Nurses on COVID-19 Vaccination

This study’s results highlighted a significant gap in nurses’ ongoing training on COVID-19 vaccination, with 70.5% of participants stating that they had not received any specific training. This situation can be explained by several factors, including the constraints imposed by the pandemic, such as workload and social distancing measures. There was also a lack of clear and regular communication about developments in vaccines and associated protocols, and, finally, the rapid introduction of RNA vaccines (Moderna, Pfizer), which did not allow nurses to train effectively. As a result, the insufficient knowledge of the specific characteristics of vaccines could have repercussions on nurses’ ability to advise and reassure patients. In comparison, countries such as China and Indonesia, where sustained training efforts have been made, report higher levels of knowledge among healthcare professionals. In China, for example, a study assessing healthcare workers (73% of whom were nurses) found that 89.2% demonstrated high knowledge levels based on a structured assessment [11]. Similarly, in Indonesia, knowledge levels among nurses were reported at 85.3% [11]. In Kenya, an assessment of healthcare professionals showed an overall knowledge score of 80% [12], while in Ethiopia, 62.5% of healthcare workers demonstrated familiarity with COVID-19 vaccines [13]. It is important to note that while our study is based on self-reported knowledge, these studies used validated assessment tests. This methodological difference should be considered when interpreting and comparing knowledge levels across studies.

### 4.2. Factors Influencing Knowledge Gaps

Historically, Morocco has performed remarkably well regarding vaccination coverage, exceeding 95% [14], and has eradicated several target diseases, according to the WHO [15]. This success is based on the accumulated experience of nurses and the ongoing training as part of the National Immunization Program [14]. However, introducing the anti-COVID-19 vaccine took place in an emergency, confronting nurses with a dual challenge: carrying out mass vaccination while ensuring the continuity of primary healthcare. Several obstacles hampered their training, including the shortage of nursing staff, with a ratio of 981 inhabitants per nurse [16]. Another relevant obstacle to continuing training for nurses in Morocco is the lack of professional recognition. The absence of promotions, pay rises, or official recognition of acquired skills reduces nurses’ motivation to continue training. In addition, primary healthcare centers lack autonomy regarding financial resources to offer appropriate training, which limits nurses’ access to learning opportunities. In addition, hierarchical procedures for reporting side effects have limited the direct coordination with pharmacovigilance centers. The transmission of reports essentially relies on provincial managers, which could explain the lack of knowledge of pharmacovigilance observed among the nurses interviewed.

### 4.3. Impact of Gaps on Communication and Acceptance of Vaccination

The nurses interviewed highlighted a lack of clear, reassuring communication with patients, which is essential to encourage them to vaccinate. Several factors hampered this communication, including the use of masks, which limited non-verbal communication; the lack of time for in-depth discussions with patients [17]; and a lack of education about the benefits and risks of COVID-19 vaccines. Morocco has set up an official website and media campaigns at the national level to raise public awareness. However, these efforts have not been enough to fill nurse communication gaps. Our results show that 57% of nurses expressed concern about the potential risks of vaccines. This skepticism is a cause for concern, as it could hinder the public support for vaccination. In Belgium, a study revealed a reluctance to vaccinate, particularly for the second dose [18]. Improving nurses’ training and reinforcing their communication about vaccines is essential to strengthen their role as role models for patients. Studies show that the more nurses know about vaccination, the more likely they are to accept and promote vaccination against COVID-19 [19].

### 4.4. Vaccination Alert and Monitoring Systems

A significant proportion of the nurses surveyed (68.5%) said their centers did not have formal alert systems to identify and report suspected COVID-19 or vaccine side effects. In addition, 76.8% of nurses had no contact with reporting centers, and 49.5% were unfamiliar with the term ’pharmacovigilance’. Although international standards encourage reporting adverse reactions, detecting them remains a challenge [20] due to the high workload of healthcare professionals, which has limited their ability to communicate in real-time to obtain advice or report adverse events (AEs) [21]. In addition, restrictive hierarchical procedures have considerably slowed down this process. Faced with this reality, nurses are expressing a strong need for training in pharmacovigilance, which is essential to ensure the effective monitoring of vaccines and to reassure the public. Ongoing training for healthcare professionals in this area is crucial to respond to the public’s concerns and ensure the accurate reporting of adverse reactions to vaccines [22].

### 4.5. Post-Vaccination Adverse Effects

Our results also revealed significant gaps in the understanding of key concepts relating to adverse drug reactions (ADRs) and post-vaccination adverse events (PVAEs) among the nurses surveyed. Although most participants stated that they were familiar with the definition of AEVRs and adverse reactions, more than half (56.5) of them could not distinguish between an adverse reaction and an adverse event. These shortcomings, partly observed in our study, are widespread among healthcare professionals, as shown by the Timor-Leste study, where knowledge scores in pharmacovigilance and ADE reporting are relatively low, particularly among nurses [23]. This confusion may adversely affect the quality of side effect monitoring, particularly in the context of vaccines, where it is crucial to categorize and identify these phenomena to guarantee patient safety correctly.

Regarding the classification of ADEs, the results show that nurses have a better grasp of classifications based on the frequency and nature of adverse reactions, which may reflect their day-to-day experience managing the known effects of drugs and vaccines. However, the poor understanding of the classification of ADEs according to their predictability suggests an urgent need for training in detecting potential adverse reactions before they occur, a key aspect in improving pharmacovigilance and preventing risks. The classification of ADEs by severity, which nurses better understand, indicates that they can better identify and react to severe adverse reactions, which is reassuring for the clinical management of patients. These results underline the need for further training for healthcare professionals, particularly in pharmacovigilance, to improve their ability to differentiate and classify adverse events correctly and thus guarantee better vaccine and drug safety.

### 4.6. Recommendations and Prospects

Several measures should be considered to enhance the healthcare system’s preparedness and responsiveness in the face of health emergencies. It is essential to expand the responsibilities of regional monitoring units by introducing a set of tasks, such as continuous nursing training, raising awareness and motivating healthcare staff, coordinating interventions, and providing effective support to professionals during crises.

At the same time, more in-service training is crucial to ensuring that new vaccines are better mastered. More targeted and regular training, tailored to the local realities of healthcare establishments, should be implemented, including face-to-face and online sessions on vaccine types, vaccination protocols, and adverse reactions. With this in mind, it would be worth considering the introduction of accelerated, targeted training courses when new vaccines are introduced, incorporating specific modules on new vaccine technologies.

In addition, developing interactive digital tools (webinars and mobile applications) could enable continuous learning without disrupting nurses’ clinical activities. Access to online learning platforms would make it easier to update knowledge and ensure that professionals are better prepared to deal with vaccine innovations.

Improving the internal communication between health authorities and healthcare professionals is essential to dispel misunderstandings and boost vaccine confidence. Closer links between nurses and pharmacovigilance centers would enable better feedback on adverse reactions and promote real-time training based on experience in the field.

Finally, a regional and local approach would allow for the identification and filling of knowledge gaps by adapting solutions to each locality’s specific needs and resources. This skills-building approach could be extended to other areas of public health, particularly chronic diseases and cancer, to ensure that nurses are properly trained and can respond effectively to current and future health challenges.

## 5. Conclusions

This study highlighted the obstacles faced by nursing professionals during the COVID-19 vaccination campaign in Morocco. Although nurses showed a remarkable commitment in the face of the pandemic, the crucial issue identified lay in the inadequacy of ongoing training, particularly in response to introducing new vaccines. This gap in the regular updating of knowledge and skills represents a potential obstacle to the effectiveness of vaccination campaigns. In addition, this study highlighted the urgent need to consolidate direct collaboration with pharmacovigilance centers to ensure the optimal management of adverse reactions and establish clear and rapid communication between healthcare professionals, which is crucial in times of a health crisis. It is essential not to interpret these challenges as a lack of preparation or competence on the part of nurses but rather as indicators of structural shortcomings in the healthcare system in the face of the unprecedented demands of a global pandemic. Therefore, strengthening ongoing training and improving the coordination between the various players in the healthcare sector is essential to responding effectively to future crises.

## Figures and Tables

**Table 1:** Demographic and professional characteristics of nursing population.

Characteristics	Participants n (%) N = 200
**Age group (years)**	
≤25	66 (33)
≥25	134 (67)
**Sex**	
Female	148 (74)
Male	52 (26)
**Training specialty**	
Versatile nurse	83 (41.5)
Midwife	49 (24.5)
Auxiliary nurse	37 (18.5)
Mental health nurse	31 (15.5)
**Length of time in the profession**	
Less than 5 years	103 (51.5)
More than 5 years	97 (48.5)
**Place of practice**	
Rural	60 (30)
Urban	140 (70)

Note: values are expressed as count (n) and percentage (%).

**Table 2:** Training, practices, resources, and perceptions related to COVID-19 vaccination among healthcare workers (N = 200).

Characteristics	Participants n (%)
**Participation in training on COVID-19 vaccination**	
No	141 (70.5)
Yes	59 (29.5)
**Implementation of systematic alerts for new patients with COVID-19**	
No	137 (68.5)
Yes	63 (31.5)
**Availability of materials for barrier measures against COVID-19**	
No	88 (44)
Yes	112 (56)
**Target population for vaccination**	
Elderly	121 (60.5)
Adults	70 (35)
Children	9 (4.5)
**Type of vaccine available in the establishment**	
Astra Zeneca	175 (87.5)
Sinopharm	144 (72)
Sputnik	161 (80.5)
Pfizer/Biotech	62 (31)
Johnson	4 (2)
Covishield	3 (1.5)
Moderna	2 (1)
Sinovac	1 (0.5)
**Knowledge of the nature of the Astra Zeneca and Johnson vaccine**	
Yes	80 (40)
No	120 (60)
**Knowledge of the nature of the Moderna vaccine**	
Yes	76 (38)
No	124(62)
**Knowledge of the nature of the Pfizer vaccine**	
Yes	82 (41)
No	118 (59)
**Perceived health risk of COVID-19 vaccine**	
Yes	114 (57)
No	86(43)

Note: values are expressed as count (n) and percentage (%).

## Data Availability

The datasets are available from the corresponding author. Please contact the corresponding author.

## Abbreviations

ADE	Adverse drug events
AE	Adverse events
COVID-19	Coronavirus Disease 19
PIE	Post-immunization events
PVAE	Post-vaccine adverse events
RNA	Ribonucleic Acid
WHO	Word Health Organization
